# The effect of different lumbar belt designs on the lumbopelvic rhythm in healthy subjects

**DOI:** 10.1186/1471-2474-15-307

**Published:** 2014-09-19

**Authors:** Christian Larivière, Jean-Maxime Caron, Richard Preuss, Hakim Mecheri

**Affiliations:** Occupational Safety and Health Research Institute Robert-Sauvé (IRSST), 505, boul. De Maisonneuve Ouest, Montreal, Quebec H3A 3C2 Canada; School of Physiotherapy and Occupational Therapy, McGill University, Montreal, Quebec J1K 2R1 Canada; Centre for Interdisciplinary Research in Rehabilitation of Greater Montreal (CRIR), Montreal, Canada

**Keywords:** Lumbar support, Coordination, Kinematics

## Abstract

**Background:**

Research suggests that in some patients with low back pain, lumbar belts (LB) may derive secondary prophylactic benefits. It remains to be determined, however, which patients are most likely to benefit from prophylactic LB use, and which LB design is optimal for this purpose. The objective of this study was to determine the effect of different lumbar belts designs on range of motion and lumbopelvic rhythm.

**Methods:**

Healthy subjects (10 males; 10 females) performed five standing lumbar flexion/extension cycles, with knees straight, during a control (no belt) and four lumbar belt experimental conditions (extensible, with and without dorsal and ventral panels; non-extensible). Motion of the pelvis and lumbar spine was measured with 3D angular inertial sensors.

**Results:**

The results suggest that adding dorsal and ventral panels to an extensible LB produces the largest lumbar spine restrictions among the four tested lumbar belt designs, which in turn also altered the lumbopelvic rhythm. On a more exploratory basis, some sex differences were seen and the sex × experimental condition interaction just failed to reach significance.

**Conclusions:**

LB may provide some biomechanical benefit for patients with low back disorders, based on the protection that may be provided against soft tissue creep-based injury mechanisms. More comprehensive assessment of different LB designs, with additional psychological and neuromuscular measurement outcomes, however, must first be conducted in order to produce sound recommendations for LB use. Future research should also to take sex into account, with sufficient statistical power to clearly refute or confirm the observed trends.

**Electronic supplementary material:**

The online version of this article (doi:10.1186/1471-2474-15-307) contains supplementary material, which is available to authorized users.

## Background

While lumbar belts (LB) do not appear to reduce the risk of a first episode of low back pain[1, 2], some patients with low back pain may derive secondary prophylactic benefits from LB use[1]. It remains to be determined, however, which patients are most likely to benefit from prophylactic LB use, and which LB design is optimal for this purpose.

Psychological[3], neuromuscular and biomechanical[4] mechanisms have all been proposed to explain the clinical benefits of LB, but remain unproven. Psychological benefits are likely associated with the perceived mechanical support derived from the LB[3], while any neuromuscular likely involves mechanisms that influence lumbar stability, such as lumbar proprioception, trunk muscle feedforward and reflex activity[5–8]. The direct biomechanical benefits of LB, on the other hand, are likely related to the mechanical stiffness of the LB, leading to decreased lumbar range of motion (ROM)[3, 4, 9], reduced stresses in the passive tissues of the posterior lumbar spine[10, 11] and potentially to reduced compressive loading of the lumbar spine[12]. The direct biomechanical impact of LB is the focus of the current study. Specifically, we aim to assess the influence of LB stiffness on lumbar spine ROM and on the coordination between the pelvis and the lumbar spine during movement, hereafter called the lumbopelvic rhythm.

To the authors’ knowledge, three previous studies have assessed body segment kinematics during lifting, while wearing a LB[9, 13, 14]. One of these studies only addressed body position at peak compression force, and therefore provides little insight into the effects of LB on movement coordination[14]. The other two studies, however, provide evidence of altered inter-segmental coordination compared to lifting without a LB, suggesting that LB use changes the natural style of lifting[9, 13]. Only McGorry and Hsiang[13], however, treated the lumbar spine and pelvis as separate segments. Their results showed similar ROM measures for two types of LB (elastic and rigid), with a decrease in lumbar flexion compensated for by an increase in pelvic flexion. No significant change in the lumbopelvic rhythm, however, was found for any of the lifting and lowering stages. Unfortunately, these authors did not provide a detailed description of the LBs used in the study, making the interpretation and application of these findings difficult. Furthermore, the LB tension was volunteer-selected, which may have affected the outcomes. Further study of the effect of different LB designs on the lumbopelvic rhythm, therefore, is necessary to enhance the knowledge in this field, and ultimately to guide the prescription of LB by healthcare practitioners.

The objective of the current study was to determine the effect of different LBs designs on the lumbopelvic rhythm of healthy male and female subjects during a forward bending task. Two broad categories of LBs were studied - categorized as extensible (elastic) and non-extensible - having first been identified as flexible enough to be used at work. Furthermore, as many commercial LB also allow the possibility of adding dorsal and/or ventral inserts, which is purported to enhance lumbar stiffness, these designs were also included. A standardized, maximal trunk flexion/extension task (without lifting) was used to reduce any variability in movement patterns associated with lower and upper limb movements, with the goal of better isolating the intrinsic effect of a LB on the lumbopelvic rhythm. Sex effects were also investigated, as differences have been previously found between males and females for both pelvic and lumbar ROM[15, 16] and lumbopelvic rhythm[17] during trunk flexion/extension tasks.

## Methods

### Subjects

Twenty healthy subjects (10 men + 10 women), aged between 18 and 65 years, were recruited on a word of mouth basis, with male and female subjects matched for age (Table 1). Exclusion criteria were as follows: back pain in the preceding month; having a body mass index (BMI) greater than 31.5 kg/m^2^ (women) or 33 kg/m^2^ (men); prior surgery of the pelvis or spinal column; scoliosis; systemic or degenerative disease; one positive response to the Physical Activity Readiness Questionnaire[18]; history of neurological diseases or deficits not related to back pain (e.g., stroke, peripheral neuropathies, balance deficits); use of anticonvulsive, antidepressive and anxiolitic medication (use of antispasmodic, anti-inflammatory and analgesic drugs for back pain were accepted); pregnancy; claustrophobia; abnormal arterial blood pressure (hypertension). All subjects were informed about the experimental protocol and potential risks and gave written consent prior to their participation. The ethics committee of the Centre for Interdisciplinary Research in Rehabilitation of Greater Montreal (CRIR) approved the study and consent form.

**Table 1:** **Characteristics of the male and female subjects**

	Males (n = 10)		Females (n = 10)		T-test
	Mean	(SD)	Mean	(SD)	*P* value
Age (yr)	26	(8)	27	(11)	0.551
Height (m)	1.80	(0.06)	1.68	(0.07)	**0.007**
Mass (kg)	80	(13)	65	(10)	**0.024**
BMI (kg/m^2^)	25	(3)	23	(3)	0.286
Spine length*	0.449	(0.034)	0.393	(0.021)	**0.002**
Extensible belt upper edge at …†	T12 [T11-T12]		T11 [T9-T12]		/
N-extensible belt upper edge at …†	T12 [T11-T12]		T11 [T9-T12]		/
Thoracic sensor upper edge at …†	T9 [T8-T9]		T8 [T7-T9]		/

*:C7 spinous process height minus L5 spinous process height; †: spinous process touched by the upper edge of the belt or upper edge of the thoracic inertial sensor, reported as mean [min-max].

Significant P values are identified in bold characters.

### Lumbar belts investigated

**Figure 1:**
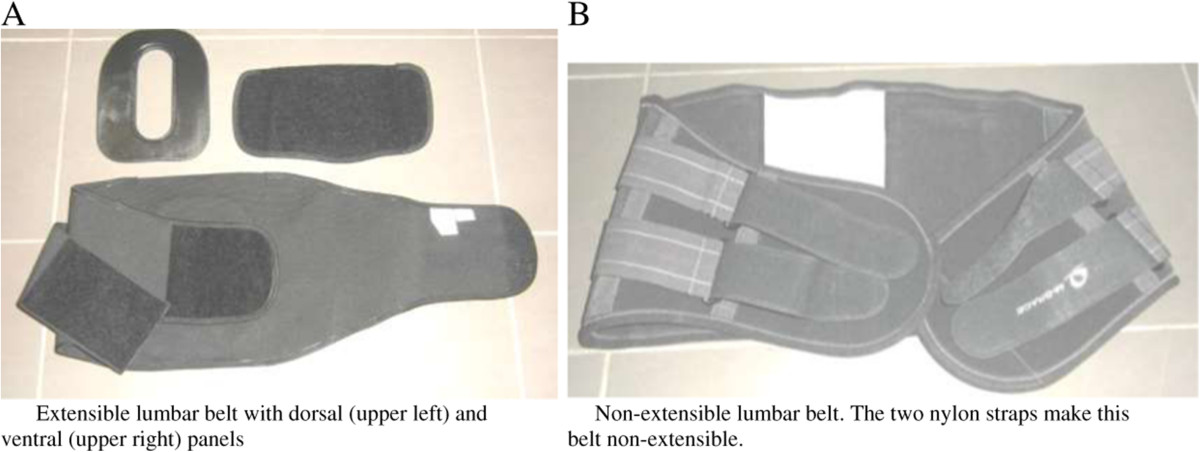
**Illustration of the tested lumbar belts.** On the left **(A)**, the extensible lumbar belt with dorsal (upper left) and ventral (upper right) panels. On the right **(B)**, the non-extensible lumbar belt. The two nylon straps make this belt non-extensible.

The two models of LB are illustrated in Figure 1, and were chosen, in consultation with an orthotist, based on functionality for use at work (flexible, comfortable), affordability and durability. The first was an extensible LB that allowed for insertion of dorsal and ventral panels (model LumboLux from Hope Orthopedic). The second was a non-extensible LB without panels (model 582 from MBrace). Both LBs had two layers of straps, secured with Velcro material; the first layer allowing for initial adjustment and placement of the LB, and the second layer (elastic material for the extensible LB and non-extensible nylon straps for the non-extensible LB) allowing the final tension adjustment. The optional ventral panel for the extensible LB was semi-rigid and covered with a Velcro material, allowing it to be anchored with the first Velcro strap. The dorsal panel was a rigid Kydex insert, with a hole in the middle to allow the spine to flex without discomfort.

Both LBs are commercially available in seven lengths, but have a standard height (front: 6 inches; back: 10 inches). The 6-inch front is typical of most “low-profile” LBs on the market, and is purported to be less restricting for trunk flexion. The use of these commercially-available LBs, therefore, did not allow for standardization of LB height based on the height of the individual subjects, which may impact the findings of this study; in particular the comparison between sexes, as women tend to be shorter than men. Consequently, the highest vertebral spinous process covered by the LB was recorded, and spine length, as defined by the distance between the L5 and C7 processes, was used as a covariate for sex comparisons (see Statistical analyses section).

**Figure 2:**
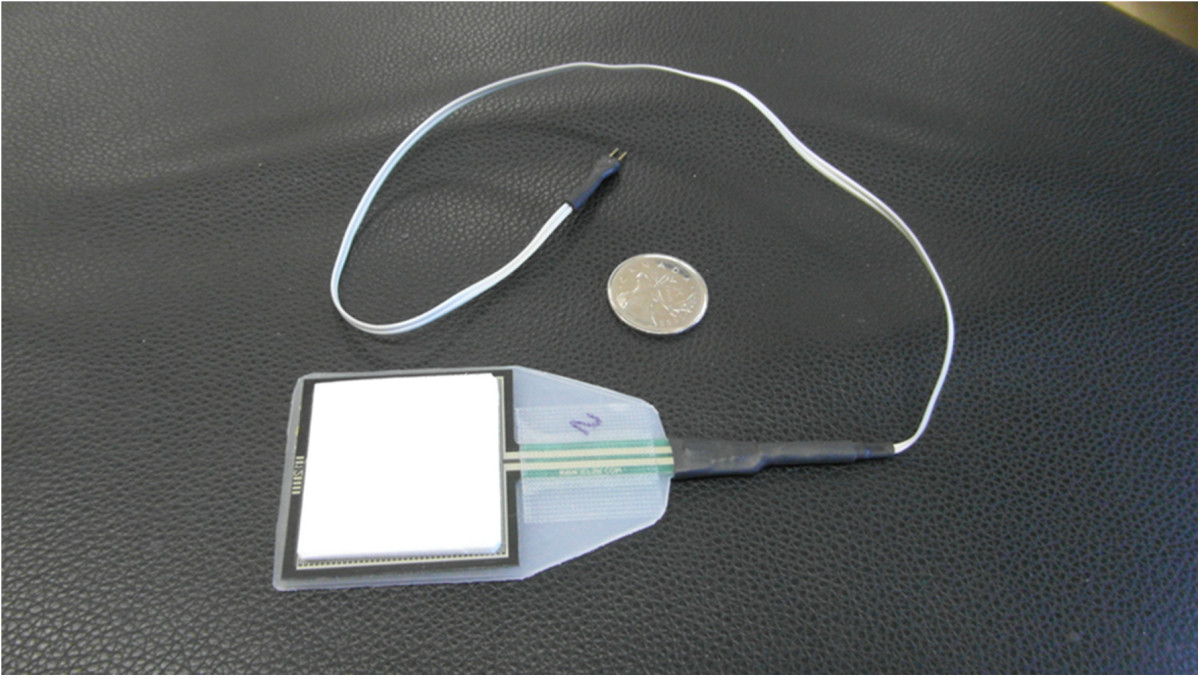
**The FSR pressure sensor allowing to standardize the tension of the lumbar belts.** To homogenize the pressure applied on the sensor, the 4.0 × 4.0 cm sensor was supported with a semi-rigid plastic sheet and covered with a Styrofoam layer (total thickness of the three layers: 2 mm).

During testing, each LB was positioned over a T-shirt, with the subject sitting, so that the lower edge of the LB covered the antero-superior iliac spines, without touching the thighs. Before each experimental condition involving a new LB, the tension of the LB was adjusted at rest, with the subject standing upright. This step was performed with the use of a FSR sensor (Force Sensing Resistor; Interlink Electronics; model FSR400; see Figure 2) fastened on the skin between the lateral aspect of the left iliac crest and the 12^th^ rib. During task performance, subjects reported that the presence of the sensor was imperceptible. Using this feedback system, the subject adjusted the tension in the LB to reach a pressure of 70 mmHg or 9.$\bar{3}$ KPa[19], allowing for a 5% error. For some smaller subjects (mostly women), however, the target pressure of 9.$\bar{3}$ KPa could not be reached. In these cases, the belt pressure was applied at a target between 8.0 and 8.8 KPa ± 5%, and a matching belt pressure was used with the subsequent male subject(s).

### Task and experimental conditions

From a standing position, keeping the knees as straight as possible during the task, subjects were asked to flex the trunk forward as far as possible without contracting the abdominals, and return to the upright position. For each of the five experimental conditions (detailed below), five consecutive cycles of the movement were performed, following the pace of a metronome (4 s to flex, 4 s to relax, 4 s to extend, 4 s to relax). The head was fully flexed through the whole task to prevent movement of the cervical vertebrae. Subjects performed the task during five randomly-ordered experimental conditions: (1) the control condition without LB (Control), (2) extensible LB without panels (ExtLB), (3) extensible LB with dorsal panel (ExtLB-D), (4) extensible LB with dorsal and ventral panels (ExtLB-DV), (5) non-extensible LB (NExtLB). These experimental conditions were designed to progressively increase the stiffness provided by LBs.

### Measurement techniques and procedures

**Figure 3:**
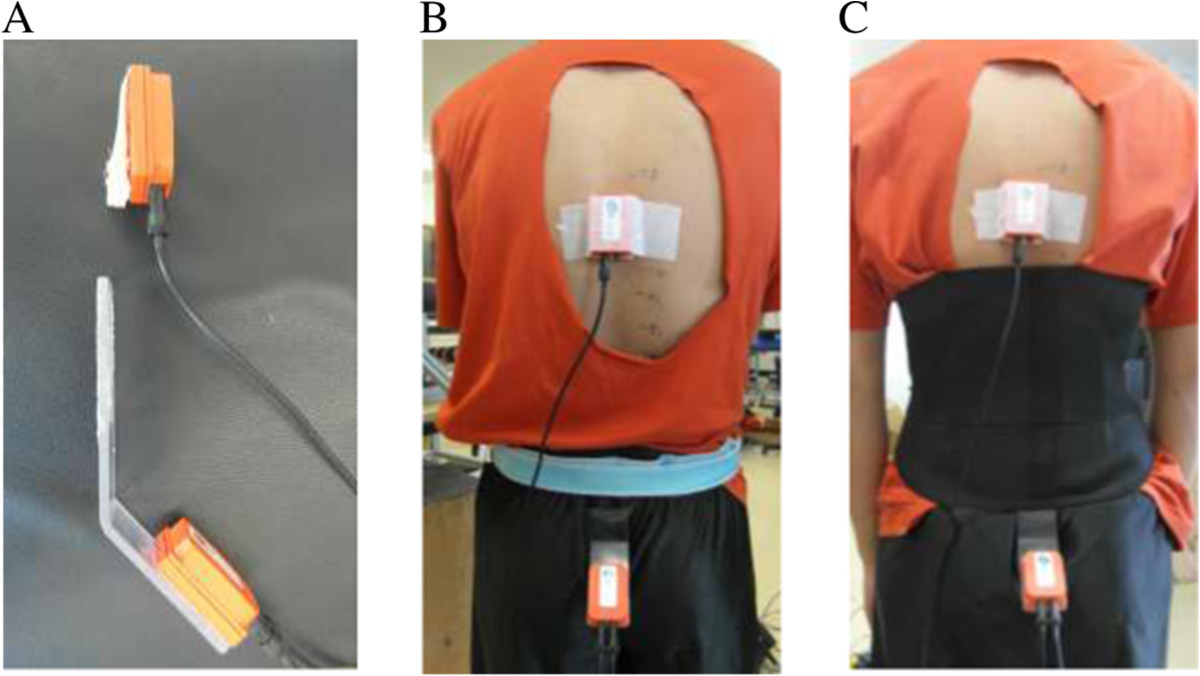
**Procedure used to allow the monitoring of the pelvis and lumbar spine without interference from the lumbar belts.** The upper inertial sensor, positioned on the thoracic spine, was secured on a piece of foam **(A)**, allowing the wire not to interfere with the LB. The lower inertial sensor was glued on an angulated piece of plastic **(A)**, which was inserted in a hole just below the short pants elastic band **(B)** and secured on the sacrum by three means (glue, Hypafix tape, 3-cm wide elastic band surrounding the sacrum and antero-superior iliac spines). The elastic band was deemed required during the control (no LB) condition and could not provide any lumbar support. The LBs were simply overlying the 3-cm elastic band, the plastic plate and the sacrum **(C)**, which prevented any discomfort during the trials. Consent to publish was given by the model in this image.

The angular kinematics of the pelvis and lumbar spine was recorded (sampling rate: 100 Hz) with a 3D-motion system comprising inertial sensors (X-Sens Motion Technologies, Enschede, The Netherlands). A first sensor followed the motion of the sacrum while a second sensor was positioned on the thoracic vertebrae not covered by the LBs, as illustrated in Figure 3. The highest spinous process covered by the thoracic sensor (generally covers two spinous processes) was identified and recorded.

### Data processing and analyses

All angular position signals recorded in the sagittal plane were first filtered using a fourth-order, zero-lag Butterworth filter with 3-Hz cut-off frequency. The five flexion/extension cycles were then separated into individual cycles and phases (flexion, extension), using a threshold of 5% of the thoracic sensor peak angular velocity.

The angular position of the sensor at the sacrum/pelvis (*Ang*_*PE*_) and on the thorax (*Ang*_*TH*_) were used to calculate the lumbar spine angle (*Ang*_*LU*_ = *Ang*_*TH*_ - *Ang*_*PE*_). *Ang*_*PE*_ and *Ang*_*LU*_ values at the beginning and end of trunk flexion were used to compute the range of motion (ROM) of the pelvis (*ROM*_*PE*_) and lumbar spine (*ROM*_*LU*_), respectively. *ROM*_*PE*_ and *ROM*_*LU*_ were summed to calculate the total trunk ROM (*ROM*_*Tot*_). The relative contribution of the lumbar spine to the total trunk ROM was then computed as follows:

$$\%\;\mathrm{RO}M_{\mathrm{LU}}=\left(\frac{\mathrm{RO}M_{\mathrm{LU}}}{\mathrm{RO}M_{\mathrm{Tot}}}\right)\times 100$$

Statistical analyses of these data showed that the *%ROM*_*LU*_ captures the same information as the corresponding variable computed for the pelvis (*%ROM*_*PE*_) or as the popular lumbopelvic ratio (*ROM*_*LU*_/*ROM*_*PE*_). The lumbopelvic ratio, however, may produce outliers when the denominator is close to zero. To provide clear and concise results, therefore, only the analysis of the *%ROM*_*LU*_ is reported as a measure of relative segmental motion.

Each phase of the motion task (flexion, extension) was further separated into four intervals (0-25; 25-50; 50-75; 75-100% of *ROM*_*Tot*_), and the intervals for the extension phase were inverted to allow for direct comparison with the flexion phases. Intervals 1 and 4, therefore, correspond to the upright and flexed positions respectively, for both phases.

The coordination between the pelvis and lumbar spine was quantified using a relative phase angle (RPA) analysis, in which the phase difference between the lumbar and pelvic segments is determined based on the velocity profiles of each segment as a function of its relative angular position[20, 21]. New standards to compute these analyses were followed[22]. A difference between the two segments of 0° indicates that the lumbar spine and pelvis segments are moving perfectly in phase, positive values indicate that the lumbar spine is leading the pelvis in the phase space, negative values indicate that the lumbar spine is lagging behind the pelvis, and 180° indicates that the segments are perfectly out of phase. Three variables were extracted from the relative phase angle curve for both the flexion and extension phases of the cycle; *RPA*_*Mean*_, *RPA*_*Std*_ and *RPA*_*Max*_, which are the mean, the standard deviation (variability of the coordination) and the extreme values (maximum when positive and minimum when negative) of the relative phase angle curve, respectively.

### Statistical analyses

All statistical analyses were done with NCSS software (version 6.0 for Windows), using a significance level (alpha) of 0.05. Because some variables showed abnormal distributions, we elected to systematically transform all variables[23] to normalize their distributions, as verified with the Wilk-Shapiro test, thus allowing the use of parametric statistical analyses. Note, however, that values reported in tables and figures are the untransformed values.

Preliminary analyses (repeated measures ANOVAs) revealed that the first movement cycle was significantly different than some of the remaining cycles (cycle 2 and/or 3 and/or 4 and/or 5) for many kinematic variables and pressure measures, possibly because of learning or some repositioning of the sensors or LBs during the first cycle. In other words, cycles 2, 3, 4 and 5 were always non-significantly different. Consequently, for each variable, the average value of the last four cycles was retained for further analyses.

Two-way ANOVAs (2 SEX × 5 CONDITION) for repeated measures on the CONDITION factor (1 control and 4 LB conditions) were carried out on all dependent variables except *%ROM*_*LU*_, for which a 3-way ANOVA (2 SEX × 5 CONDITION × 4 INTERVAL) for repeated measures on the CONDITION and INTERVAL (0-25; 25-50; 50-75; 75-100% of *ROM*_*Tot*_) factors was used. Separate analyses were performed for the flexion and extension phases. Post-hoc pairwise comparisons were carried out with the Tukey-Kramer test.

To determine whether sex comparisons could be confounded by the number of vertebrae covered by the LBs (for variables specific to the lumbar spine), ANCOVAs were carried out for each condition (for all dependent variables) and interval (for *%ROM*_*LU*_ only), using spine length as a covariate.

## Results

### Assessment of potential confounding variables

The area covered by the LB in males and females is described in Table 1, confirming that LBs covered more vertebrae in women, which in turn forced the positioning of the upper inertial sensor on higher thoracic vertebrae. However, on average, the difference was only one vertebra. These results are explained by the significantly smaller spine length of females (39.3 ± 2.1 cm) relative to males (44.9 ± 3.4 cm).

The pressure generated by the belt during the upright and flexed trunk postures, as well as during the entire task (last four cycles), did not show SEX main effects, although a SEX × CONDITION interaction reached significance in the upright position (Table 2). Women showed a decrease of pressure across the belt conditions (from C2 to C5) whereas the opposite was seen in males, although these changes were not significantly different (1-way ANOVAs for each sex separately). However, this led to a significant SEX effect during the NExtLB condition (Δ = 0.95 KPa; *P* = 0.027), as further disclosed with separate T-tests. This might be explained by the fact that even though we paired the belt pressures of some men with those of smaller women, the difference in belt pressure between sexes increased as the stiffness of the belt increased since the belt could adapt less to the body surface. A stiffer LB would prevent the belt from wrapping tightly around the abdomen of smaller women relatively to male subjects, which were bigger in general.

**Table 2:** **Lumbar belt pressure values (KPa) corresponding to different trunk postures and statistical analyses**

Trunk posture	Sex	Mean (SD) values for each experimental condition				ANOVA (P values)			Post hoc (C)
		ExtLB (C2)	ExtLB-D (C3)	ExtLB-DV (C4)	NExtLB (C5)	Sex (S)	Condition (C)	S × C	
Upright	♂	8.85 (0.78)	8.91 (0.77)	9.09 (0.64)	9.29 (0.58)	0.172	0.993	**0.008**	/
	♀	8.86 (0.62)	8.74 (0.79)	8.62 (0.70)	8.34 (1.09)				
Flexed	♂	8.93 (0.55)	9.00 (0.40)	8.93 (0.46)	8.53 (0.55)	0.188	**< 0.001**	0.976	C2,C3,C4 > C5
	♀	8.60 (0.61)	8.55 (0.78)	8.57 (0.60)	8.19 (0.80)				
Mean across	♂	8.89 (0.67)	8.96 (0.61)	9.01 (0.56)	8.91 (0.68)	0.160	0.168	0.140	/
Entire task	♀	8.73 (0.62)	8.65 (0.79)	8.60 (0.65)	8.27 (0.96)				

♂: males; ♀: females; LB: lumbar belt; ExtLB: extensible LB without panels; ExtLB-D: extensible LB with the dorsal panel; ExtLB-DV: extensible LB with dorsal and ventral panels; NExtLB: non-extensible LB. Significant P values are identified in bold characters.

Although no significant difference was seen in the upright position, the NExtLB condition showed a significantly lower pressure (8.36 KPa) than other belt conditions (8.76 to 8.78 KPa) in the flexed position (Table 2). This may be explained by a pouch created at the position of the pressure sensor, which likely corresponded to the space between the straps of the NExtLB. However, although statistically significant, this difference was small (4.6%) and considered negligible.

### Effect of experimental conditions (LB designs) on ROM

**Figure 4:**
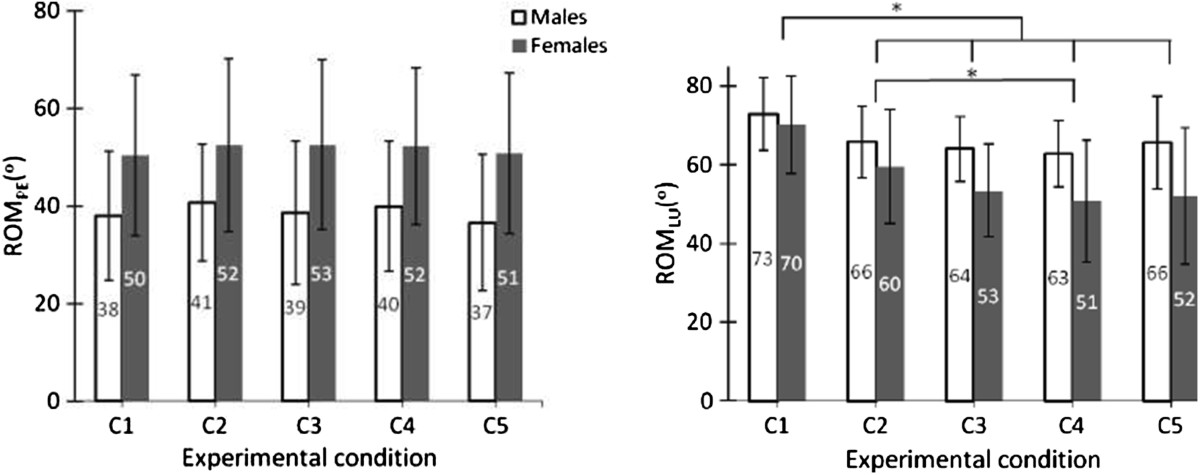
**Range of motion of the pelvis (** ***ROM***_***PE***_ **) and lumbar spine (** ***ROM***_***LU***_ **) in males and females for each lumbar belt (LB) experimental condition.** C1 (control): no LB; C2 (ExtLB): extensible LB without panels; C3 (ExtLB-D): extensible LB with the dorsal panel; C4 (ExtLB-DV): extensible LB with dorsal and ventral panels; C5 (NExtLB): non-extensible LB. *Statistically significant differences between experimental conditions are identified at the top of each graph.

The SEX × CONDITION interactions were not statistically significant (Table 3), although a trend (*P* = 0.071) was observed for *ROM*_*LU*_ (Figure 4). SEX was significant only for *ROM*_*PE*_, with females showing a 13° higher ROM than males (52° > 39°). For *ROM*_*LU*_, accounting for sex differences in spine length with the use of ANCOVAs (for each experimental condition) did not change the conclusions (no SEX effect; spine length covariate was not significant).

**Table 3:** **Statistical results (** ***P*** values*) corresponding to the effect of sex and experimental conditions on range of motion (ROM) variables during the flexion phase

Variable	ANOVA (P values)			Post hoc (C)
	Sex (S)	Condition (C)	S × C	
*ROM*_*PE*_	**0.049**	0.332	0.790	/
*ROM*_*LU*_	0.122	**< 0.001**	*0.071*	C1 > C2,C3,C4,C5; C2 > C4

LB: lumbar belt; C1 (control): no LB; C2 (ExtLB): extensible LB without panels; C3 (ExtLB-D): extensible LB with the dorsal panel; C4 (ExtLB-DV): extensible LB with dorsal and ventral panels; C5 (NExtLB): non-extensible LB. *Significant *P* values are identified in bold characters, while trends (0.05 < *P* < 0.10) are identified in italics.

Wearing a LB did not influence *ROM*_*PE*_ but affected *ROM*_*LU*_ (Table 3). More precisely, wearing any LB decreased *ROM*_*LU*_ relative to the control condition by an amount ranging from 9 to 15° (Figure 4). Considering that *ROM*_*PE*_ was not affected, *ROM*_*Tot*_, represented by the sensor positioned at the thoracic level, also significantly decreased (ANOVA *P* < 0.001; Post hoc comparisons: C1 > C3,C4,C5 and C2 > C5) by an amount ranging from 6 to 13°. Also, the ExtLB-DV condition limited more *ROM*_*LU*_ than the ExtLB condition.

### Effect of experimental conditions (LB designs) on the lumbopelvic rhythm variables

**Figure 5:**
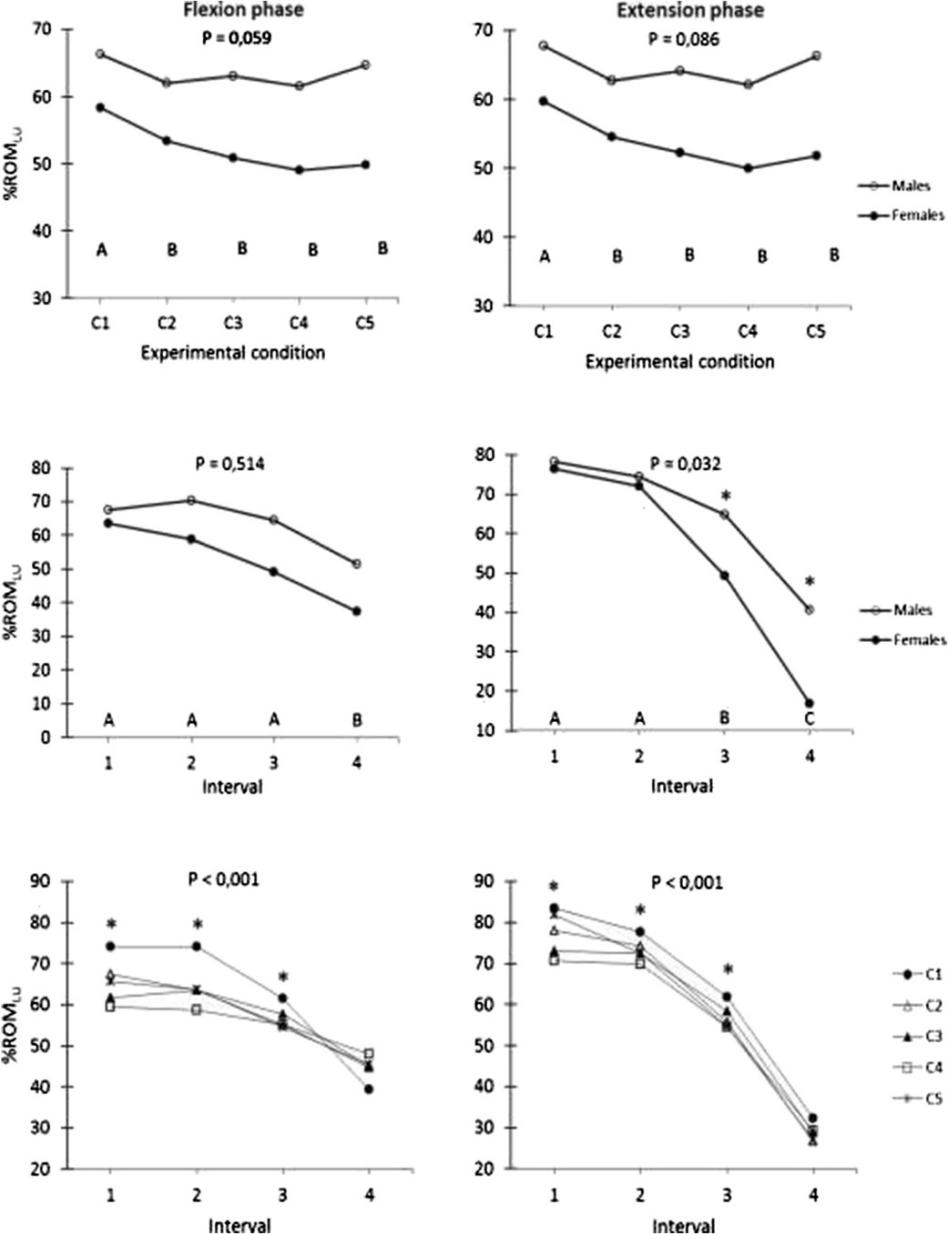
**The relative contribution of the lumbar spine to the total trunk ROM (** ***%ROM***_***LU***_ **variable), illustrated in different plots to understand the SEX × CONDITION (upper plots), SEX × INTERVAL (middle plots) and CONDITION × INTERVAL (lower plots) interactions.** *P* values reported at the top of each plot correspond to the shown interaction. Left and right plots are for the flexion and extension phases, respectively. Please keep in mind that the extension interval values were inverted to allow comparisons with the flexion phase intervals. Experimental conditions (upper plots) or intervals (middle plot) that were different (*P* < 0.05) are identified with different letters (here A, B and sometimes C) at the bottom of the corresponding graphs. When the interaction was significant, asterisks (*) were positioned to indicate where SEX differences for a specific interval (right middle plot) or CONDITION differences for a specific interval (lower plots) were significant according to post hoc analyses. C1 (control): no LB; C2 (ExtLB): extensible LB without panels; C3 (ExtLB-D): extensible LB with dorsal panel; C4 (ExtLB-DV): extensible LB with dorsal and ventral panels; C5 (NExtLB): non-extensible LB. Standard deviations were not shown for clarity.

The *%ROM*_*LU*_ variable, computed across phases (flexion, extension) and intervals, revealed that all main effects (SEX, CONDITION, INTERVAL) and some interactions reached statistical significance (Table 4). The ANCOVAs carried out to account for spine length differences between sexes did not uncover any difference relative to sex effects for this variable. SEX × CONDITION interactions just failed to reach statistical significance (0.05 < *P* < 0.10) but a SEX × INTERVAL (during extension) and CONDITION × INTERVAL interaction reached significance. Details of these findings are illustrated in Figure 5. Table 5 provides additional statistical explanations of the CONDITION × INTERVAL interactions based on separate one-way ANOVAs for repeated measures between conditions for each interval and between intervals for each condition. For space constraints, these results are described and immediately interpreted in details in the discussion.

**Table 4:** **Statistical results (** ***P*** values*) corresponding to the effect of sex, experimental conditions and intervals on the relative contribution of the lumbar spine to the total trunk range of motion (%ROM_**LU**_ **)**

Phase	ANOVA (*P* values)						
	Sex (S)	Condition (C)	Interval (I)	S × C	S × I	C × I	S × C × I
Flexion	**0.024**	**<0.001**	**<0.001**	*0.059*	0.514	**<0.001**	0.444
Extension	**0.041**	**<0.001**	**<0.001**	*0.086*	**0.032**	**<0.001**	0.935

*Significant *P* values are identified in bold characters, while trends (0.05 < *P* < 0.10) are identified in italics.

**Table 5:** **Statistical results (** ***P*** values*) corresponding to the post-hoc comparisons required to explain the CONDITION × INTERVAL significant interactions obtain for the %ROM_**LU**_ **variable**

	Flexion phase		Extension phase	
	ANOVA	Post-hoc comparisons	ANOVA	Post-hoc comparisons
	P value	(Tuckey-Kramer)	P value	(Tuckey-Kramer)
Interval*		Between conditions		Between conditions
1	**<0.001**	1 > all; 2 > 3,4; 5 > 4	**<0.001**	1,5 > 3,4; 2 > 4
2	**<0.001**	1 > all; 2,3,5 > 4	**0.011**	1 > 4
3	**0.009**	1 > 2,4,5	**0.004**	1 > 2,4,5
4	*0.084*	/	0.135	/
Condition†		Between intervals		Between intervals
1	**<0.001**	1,2 > 3 > 4	**<0.001**	1,2 > 3 > 4
2	**<0.001**	1 > 3,4; 2 > 4	**<0.001**	1,2 > 3 > 4
3	**<0.001**	1,2,3 > 4	**<0.001**	1,2 > 3 > 4
4	**0.027**	1 > 4	**<0.001**	1,2 > 3 > 4
5	**<0.001**	1 > 3,4; 2 > 4	**<0.001**	1 > 2 > 3 > 4

*In the first part of the table, a one-way ANOVA for repeated measures on the CONDITION factor was carried out for each interval separately. † A one-way ANOVA for repeated measures on the INTERVAL factor was carried out for each condition separately. *Significant *P* values are identified in bold characters, while trends (0.05 < *P* < 0.10) are identified in italics.

**Figure 6:**
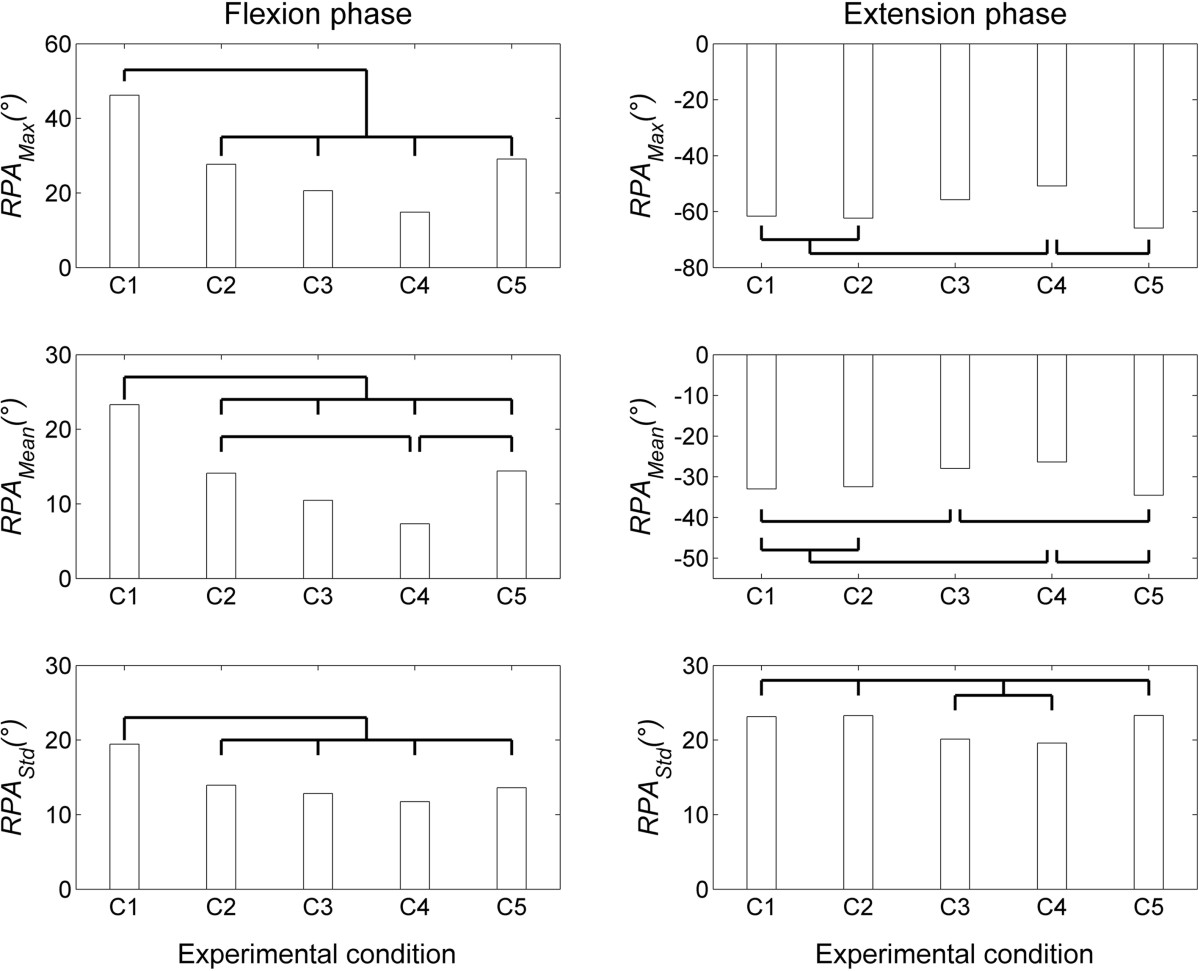
**Relative phase angle variables, all showing statistically significant differences between experimental conditions.** C1 (control): no LB; C2 (ExtLB): extensible LB without panels; C3 (ExtLB-D): extensible LB with dorsal panel; C4 (ExtLB-DV): extensible LB with dorsal and ventral panels; C5 (NExtLB): non-extensible LB. *Experimental conditions that were different (*P* < 0.05) are identified at the top of each graph.

The relative phase angle variables showed neither statistically significant SEX × CONDITION interactions, nor sex differences (Table 6), and the ANCOVAs carried out to account for spine length differences between sexes did not make any difference relative to sex effects on these variables. The CONDITION factor was significant for the three RPA variables (*RPA*_*Max*_, *RPA*_*Mean*_ and *RPA*_*Std*_) and the two phases (flexion and extension), as further illustrated in Figure 6. More specifically, during the flexion phase, the three *RPA* variables were significantly higher during the control condition than all the LB conditions. *RPA*_*Mean*_ showed further differences between the different LB designs, with the ExtLB-DV condition showing lower values than the ExtLB and NExt-LB conditions. During the extension phase, the three *RPA* variables were significantly higher during the ExtLB-DV condition than the control, the ExtLB and NExt-LB conditions. Additional differences involved the ExtLB-D condition, showing higher *RPA*_*Mean*_ values than the control and NExt-LB conditions, and showing lower *RPA*_*Std*_ values than the control, ExtLB and NExt-LB conditions.

**Table 6:** **Statistical results (** ***P*** values*) corresponding to the effect of sex and experimental conditions on relative phase angle variables

Phase angle	Phase	ANOVA (P values)			Post hoc (C)
Variable		Sex (S)	Condition (C)	S × C	
*RPA*_*Max*_	Flexion	0.592	**<0.001**	0.913	C1 > C2,C3,C4,C5
	Extension	*0.080*	**<0.001**	0.445	C4 > C1,C2,C5
*RPA*_*Mean*_	Flexion	0.546	**<0.001**	0.921	C1 > C2,C3,C4,C5 C4 < C2,C5
	Extension	0.114	**<0.001**	0.599	C4 > C1,C2,C5 C3 > C1,C5
*RPA*_*Std*_	Flexion	0.927	**<0.001**	0.871	C1 > C2,C3,C4,C5
	Extension	*0.064*	**<0.001**	0.456	C1,C2,C5 > C3,C4

LB: lumbar belt; C1 (control): no LB; C2 (ExtLB): extensible LB without panels; C3 (ExtLB-D): extensible LB with dorsal panel; C4 (ExtLB-DV): extensible LB with dorsal and ventral panels; C5 (NExtLB): non-extensible LB. *Significant *P* values are identified in bold characters, while trends (0.05 < *P* < 0.10) are identified in italics.

## Discussion

The findings of this study provide direct evidence of the biomechanical effects of LB use during trunk flexion/extension. Wearing a LB significantly reduced lumbar ROM while pelvis ROM remained unchanged. An effect of LB design was also found, with lumbar ROM being more greatly reduced by the ExtLB-DV than by the ExtLB design. LB use also significantly changed the lumbopelvic rhythm, as revealed by different coordination variables.

On a more exploratory basis, sex differences were also seen. Males showed less pelvic ROM and, in general, more contribution to overall trunk ROM from the lumbar spine (*%ROM*_*LU*_). SEX × CONDITION interactions, however, did not reach statistical significance, although some trends (0.05 < *P* < 0.10) were observed. Furthermore, only a single interaction effect was found between sex and other independent variables. Consequently, for clarity, the effect of experimental conditions and of sex will be discussed separately.

### Effect of experimental conditions (LB designs) on ROM

The first finding of note was that all LB designs significantly reduced lumbar ROM relative to the control condition, while pelvis ROM remained unchanged. This emphasizes the specific effect of LB use on lumbar ROM, and is likely explained by an increase in lumbar stiffness[24].

Previous studies have also shown a reduced lumbar ROM with LB use[3, 4, 9]. This, however, was often accompanied by an enhanced ROM at peripheral joints such as at the knees and hip, or in the thoracic spine[3, 9]. The current study, however, was specifically designed to isolate the intrinsic effect of a LB on the lumbopelvic rhythm. Pain-free subjects were used, and, unlike freestyle lifting, the selected task was limited to trunk flexion/extension. Under these strict conditions, no effect of LB use was found on pelvis ROM, which suggests that the direct mechanical effect of LBs is limited to the lumbar spine (the immediate effect on thoracic spine ROM, however, was not assessed). Furthermore, the purpose of the task being to achieve maximal trunk flexion, the possibility that a part of the reduced lumbar ROM was explained by sensory feedback serving as a reminder for good postural hygiene was excluded.

The lumbar ROM was also more affected by the ExtLB-DV than by the ExtLB design, the former showing the largest effect (15°) relative to the control condition. This suggests that adding ventral and dorsal panels further stiffens the lumbar spine, as proposed by LB manufacturers. One previous study has directly evaluated the effects of different LB designs on lumbar stiffness, using a sudden trunk perturbation task[19]. The non-extensible LB used in this previous study increased trunk stiffness by 14% relative to an extensible LB. Adding a ventral panel to the non-extensible LB, however, produced no further measureable increase in stiffness (the effect of panels with the extensible LB was not assessed). Assessing the findings of this previous study, along with the different effects of LB design on different task and outcome measures (*ROM*_*LU*_, *%ROM*_*LU*,_ relative phase angle variables) in the current study, suggests the need to consider both mechanical and neuromuscular outcome measures to better delineate the pros and cons of different LB designs.

From a clinical perspective, the decrease in lumbar ROM that was observed with all LB designs in the current study may have a positive impact on mechanisms of injury linked with the progressive creep of the lumbar spine posterior passive-tissues. Induced tissue creep, via sustained or repetitive lumbar flexion, is known to reduce intrinsic lumbar stiffness, impair back muscle reflexes[25–28] and disturb trunk postural control[29]. Tissue creep may also trigger inflammatory processes and enhance muscle spasm[30]. Cumulative exposure to repetitive or sustained trunk flexion, which is common in many occupations (e.g. manual materials handlers, roofers, bricklayers, gardeners, movers, etc.), may therefore predispose workers to low back injury and pain. A non-negligible reduction of lumbar flexion, by 9 to 15° with the use of LB may therefore reduce these risks by decreasing tissue creep. This might be particularly helpful when back muscle fatigue develops during repetitive activities, knowing that lumbar flexion progressively arises in these circumstances[31]. This may also help patients with lumbar posterior passive-tissue injuries to return to work more rapidly without exacerbating their pain or compromising their safety.

### Effect of experimental conditions (LB designs) on the lumbopelvic rhythm variables

The findings of the current study indicate that LB use may affect the coordination of movement between the lumbar spine and pelvis. As might be expected from the reduced lumbar spine ROM with LB use, the contribution of the lumbar spine to the overall trunk ROM (*%ROM*_*LU*_) was also reduced for all LB conditions, in both the flexion and extension phases (Figure 5**-** upper plots). No difference, however, was noted between any of the LB conditions. This effect is illustrated in the interval-specific results (Figure 5 – middle and lower plots). As in previous studies[32–35], the lumbar spine contributed progressively less to overall trunk flexion (*%ROM*_*LU*_) as trunk flexion increased, with the difference becoming significant in the later intervals for both the flexion (left middle plot) and extension (right middle plot) movements. During the flexion movement, however, the progressive decrease of *%ROM*_*LU*_ across intervals was less pronounced with all LB designs conditions relative to the control condition (Figure 5 – left lower plot). A similar, although less evident effect also appears during the extension movement (Figure 5 – right lower plot). In other words, the reduction of the lumbar spine’s contribution to overall trunk motion, as a result of LB use, is more pronounced when standing than when the trunk is fully flexed (Figure 5; Table 5). This more in-depth analysis also revealed some differences between specific LB designs in the more upright intervals of motion (1^st^ and 2^nd^ interval during flexion; 1^st^ interval during extension). Here, the ExtLB-DV design produced the greatest reduction in relative lumbar ROM when compared to one or more of the remaining LB designs. Once again, this is likely reflective of the additional stiffness provided by the dorsal and ventral panels in the ExtLB-DV design, leading to an altered lumbopelvic rhythm in addition to a reduction in lumbar ROM, as previously discussed.

The RPA variables (*RPA*_*Max*_, *RPA*_*Mean*_ and *RPA*_*Std*_) were all sensitive to the CONDITION factor during both flexion and extension. *RPA*_*Max*_ and *RPA*_*Mean*_ values indicated that the lumbar spine was leading the pelvis during flexion (positive values) whereas the opposite was observed during extension (negative values). However, all LB designs reduced the leading of the lumbar spine over the pelvis during flexion, which was in line with *%ROM*_*LU*_ results (Figure 5 – left lower plot). Moreover, *RPA*_*Mean*_ results showed that the ExtLB-DV produced the largest effect in this respect, leading to significant differences with the ExtLB and NExt-LB conditions. Likewise, the largest effects were observed for the ExtLB-DV condition (for *RPA*_*Max*_ and *RPA*_*Mean*_ variables) during the extension phase, but in the opposite direction (pelvis leading the lumbar spine to a lesser degree relative to the control, ExtLB and NExt-LB conditions). This was also observed for the ExtLB-D condition, but to a lesser extent (*RPA*_*Mean*_ variable only; relative to the control and NExt-LB conditions). Overall, these findings further confirm that all of the investigated LB designs altered the lumbopelvic rhythm, and that this effect was enhanced with the use of the dorsal panel, although the clinical significance of these findings remains to be determined. These findings are likely directly related to the mechanical stiffness provided by the LB, as inter-segmental coordination is inextricably linked to the control of segmental stiffness[36]. However, the use of the ventral panel, in addition to the dorsal panel, did not make a further difference.

All LB designs led to a reduction in the variability of the relative phase angle (*RPA*_*Std*_*)* between the lumbar spine and pelvis during trunk flexion, with a similar effect observed for the ExtLB-D and ExtLB-DV designs during trunk extension. While an external support may reduce the risk of injury due to a temporary loss of active segmental control (due to muscle fatigue, loss of concentration, etc.), it may also allow for less room for variability in movement patterns by the central nervous system. According to a recent hypothesis[37, 38], such a reduced variability may be detrimental for musculoskeletal health, particularly when repetitive work activities are performed, as it will lead to repetitive loading of the same anatomical structures, and potentially to mechanical tissue fatigue. Reduced motor variability, for example, has been observed in subjects with chronic pain[39], and may be emblematic of a pre-existing motor dysfunction or of an inappropriate long-term compensation following an acute injury[40].

### Effect of sex

While differences between the sexes were not the primary focus of this study, some effects of sex were observed that may be important in future work. Males showed less pelvic ROM, which concurs with previous findings[15, 17]. Unlike previous studies[15, 16], however, no difference was found in our data between men and women for lumbar spine ROM.

Fitting with the findings above, the male participants in our study showed more contribution from the lumbar spine (*%ROM*_*LU*_) during trunk extension, particularly in the flexed trunk posture. This is in line with the findings of Nelson-Wong et al.[17], who showed a higher lumbar/hip ratio in males during extension from trunk flexion. While our analysis suggests that spine length differences did not drive these sex effects, certain SEX × CONDITION interactions did near statistical significance (0.05 < *P* < 0.10) for *ROM*_*LU*_ and *%ROM*_*LU*_ (see Figure 5 – upper plots) variables. This suggests that future studies should account for sex, as these differences may become more apparent in studies conducted with larger sample sizes.

### Perspectives

Although current evidence suggests that LB use does not reduce the risk of a first episode of low back pain[1, 2], there may be a role for LBs for secondary and tertiary prevention, as suggested by a systematic review showing more controversial findings for patients with low back pain[1] and the positive clinical findings observed in the more recent randomized clinical trials (RCTs)[41, 42]. Interestingly, these RCTs were of longer duration (>3 months). More research is needed to determine which workers with low back pain will benefit more from this type of conservative intervention.

The results of the present study show that LB use, for all designs tested, leads to a reduction in lumbar ROM. This suggests that LB may be a good short or long-term solution for patients with low back disorders, based on the protection that may be provided against soft tissue creep-based injury mechanisms[30]. Our results also suggest that adding dorsal and ventral panels to an extensible LB produces the largest restrictions to lumbar spine motion, among the four tested LB designs and the largest alterations in the relative amplitude (*%ROM*_*LU*_) and RPA (*RPA*_*Max*_ and *RPA*_*Mean*_) measures of the lumbopelvic rhythm. LB use, however, also led to a reduction in the variability of segmental coordination patterns (*RPA*_*Std*_). This may be viewed as a negative effect, in light of current hypotheses related to the protective effects of motor variability against tissue fatigue and over-use injury, and suggests that LB are a poor long term solution for patients. It is thus difficult to give clear recommendations for LB use, and even trickier to propose a specific LB design, based on the findings of this study. A more comprehensive assessment of different LB designs, with the use of different neuromuscular measurement outcomes, must still be conducted, however, to produce more individualized recommendations for LB use. Longitudinal studies must also be conducted to determine any long-term effects of LB use, taking into account any possible central nervous system adaptations, and their effect on neuromuscular[43, 44], psychological (e.g. fears of pain/movement; not tested so far) and clinical[1, 41, 42] outcomes.

The identification of patients that benefit more from the use of LBs during clinical trials would demonstrate that the net clinical benefits (pain, disability) of this simple intervention might outweigh the hypothesized but not yet supported detrimental effects feared by some clinical practitioners (false sense of security, psychological dependence, maladaptative neuromuscular adaptations, muscle atrophy and weakness). In the interim, the cautious practitioner might prescribe a LB on working days on which the patient had, or expected that they might develop, low back pain[42]. This is in line with the recommendation of not using a LB over several consecutive days or weeks[45]. Such a prescription might also be accompanied with clear messages about the importance of trunk muscle support, the beneficial effect of physical/work activity for the low back, and as such that activities involving the low back must be progressively resumed as soon as possible when symptoms decrease, and especially when the LB is withdrawn. Key messages provided in the “Backbook” leaflet[46] would be recommended in this perspective.

### Strengths and limitations of the study

This study comprises several strengths. Firstly, great care was taken to minimize any potential interference between the LBs and the kinematic sensors and to control for LB pressure. The only potential confounding variable, which was related to the relative height of the LBs, was statistically accounted for to make valid sex comparisons. Secondly, a comprehensive study of the lumbopelvic rhythm was provided with the use of complementary kinematic analyses, as discussed above. Third, more than two LB designs were contrasted. Finally, we used a standardized task that allowed us to isolate the intrinsic effect of a LB on the lumbopelvic rhythm.

Limitations must also be acknowledged. These results cannot be generalized to people with back pain. Furthermore, our analysis did not account for any effect on the lumbothoracic rhythm. Finally, this exploratory study of the possible effect of sex was likely underpowered, and as such we encourage further testing of these findings with a larger sample size.

## Conclusions

LB may provide some biomechanical benefit for patients with low back disorders, based on the protection that may be provided against soft tissue creep-based injury mechanisms. A more comprehensive assessment of different LB designs, with the use of different psychological and neuromuscular measurement outcomes, however, must be conducted to more fully understand the effects of LB use, before more targeted recommendations for LB use (or avoidance) can be produced for patient subgroups. Future research should also take sex into account, with sufficient statistical power to clearly refute or confirm the observed trends.

## Authors’ original submitted files for images

Below are the links to the authors’ original submitted files for images. Authors’ original file for figure 1 Authors’ original file for figure 2 Authors’ original file for figure 3 Authors’ original file for figure 4 Authors’ original file for figure 5 Authors’ original file for figure 6

## Abbreviations

*Ang*_*LU*_: Lumbar spine angle
*Ang*_*PE*_: Pelvis angle
*Ang*_*TH*_: Thorax angle
ANCOVA: Analysis of covariance
ANOVA: Analysis of variance
BMI: Body mass index
C1 to C5: Experimental conditions: control (C1), ExtLB (C2), ExtLB-D (C3), ExtLB-DV (C4) et NExtLB (C5)
ExtLB: Extensible LB without panels
ExtLB-D: Extensible LB with dorsal panel
ExtLB-DV: Extensible LB with dorsal and ventral panels
LB: Lumbar belt
NExtLB: Non-extensible LB
RCT: Randomized clinical trial
ROM: Range of motion
*ROM*_*PE*_: ROM of the pelvis
*ROM*_*LU*_: ROM of the lumbar spine
*%ROM*_*LU*_: Relative contribution of the lumbar spine to *ROM*_*Tot*_
*ROM*_*Tot*_: Total trunk ROM
RPA: Relative phase angle
*RPA*_*Max*_: Extreme values of the RPA curve
*RPA*_*Mean*_: Mean value of the RPA curve
*RPA*_*Std*_: Standard deviation value of the RPA curve
