# Supplementary figures and images for: The effect of different lumbar belt designs on the lumbopelvic rhythm in healthy subjects
Source: BMC Musculoskelet Disord. 2014 Sep 19;15:307. doi: 10.1186/1471-2474-15-307 (PMC4190283; doi:10.1186/1471-2474-15-307)

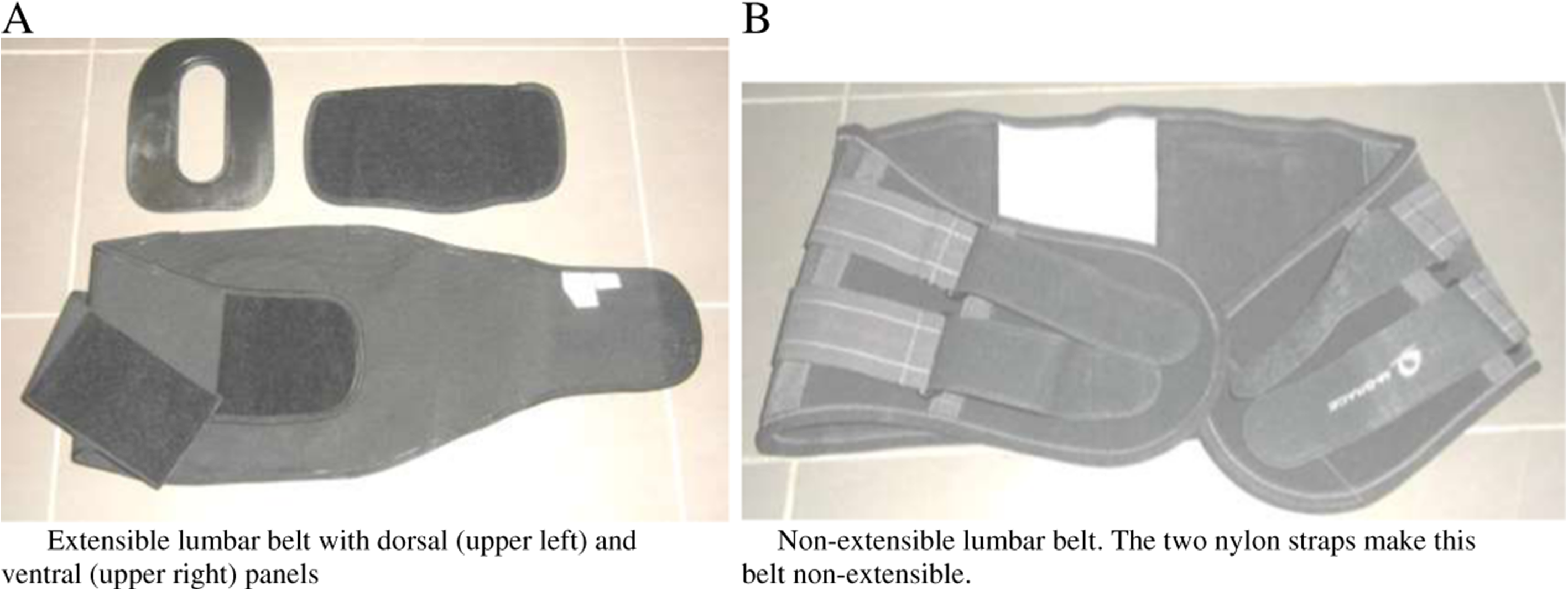

Supplement: Supplementary file 1 — Authors’ original file for figure 1 [file 12891_2014_2260_MOESM1_ESM.tif]

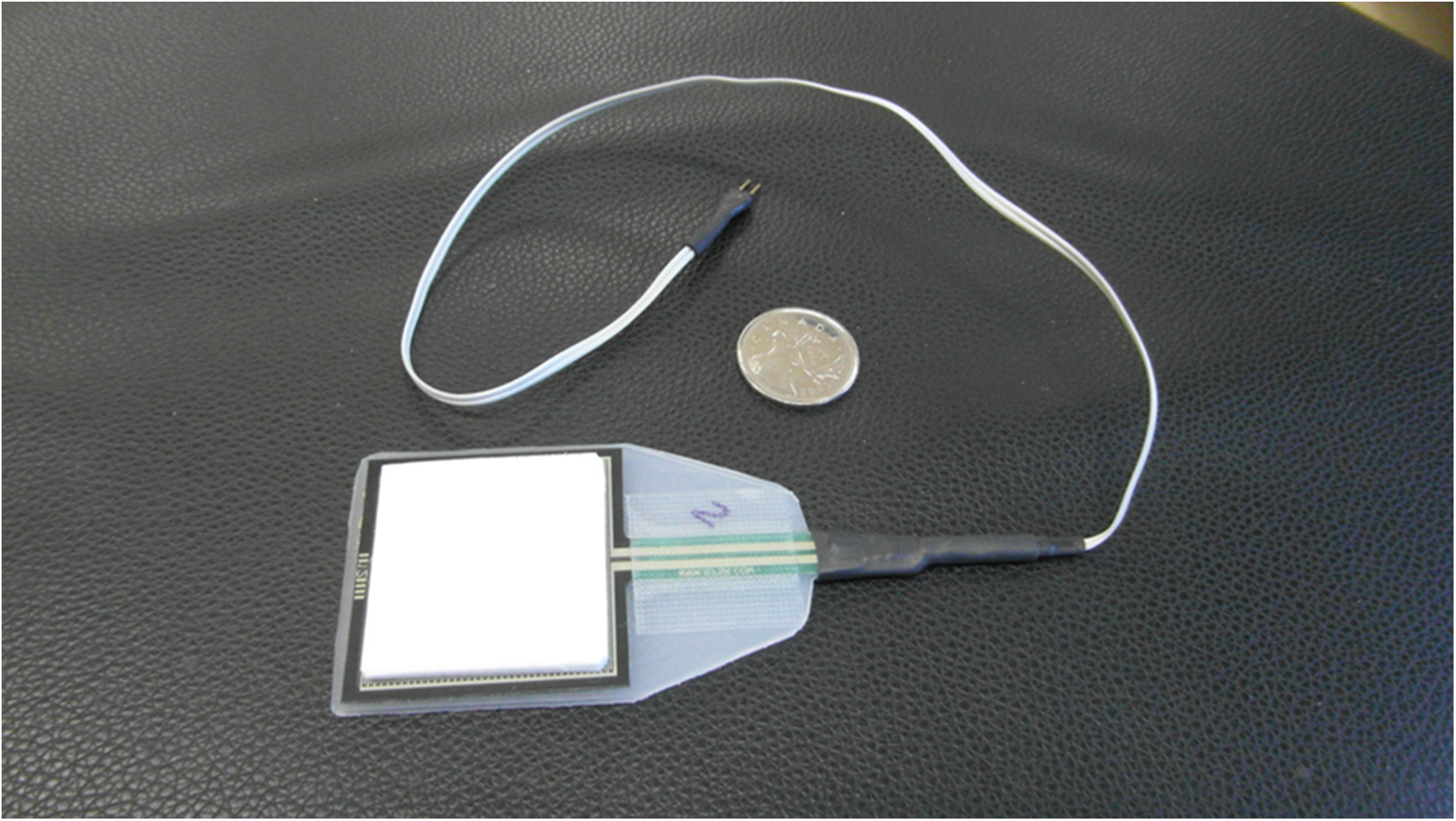

Supplement: Supplementary file 2 — Authors’ original file for figure 2 [file 12891_2014_2260_MOESM2_ESM.tif]

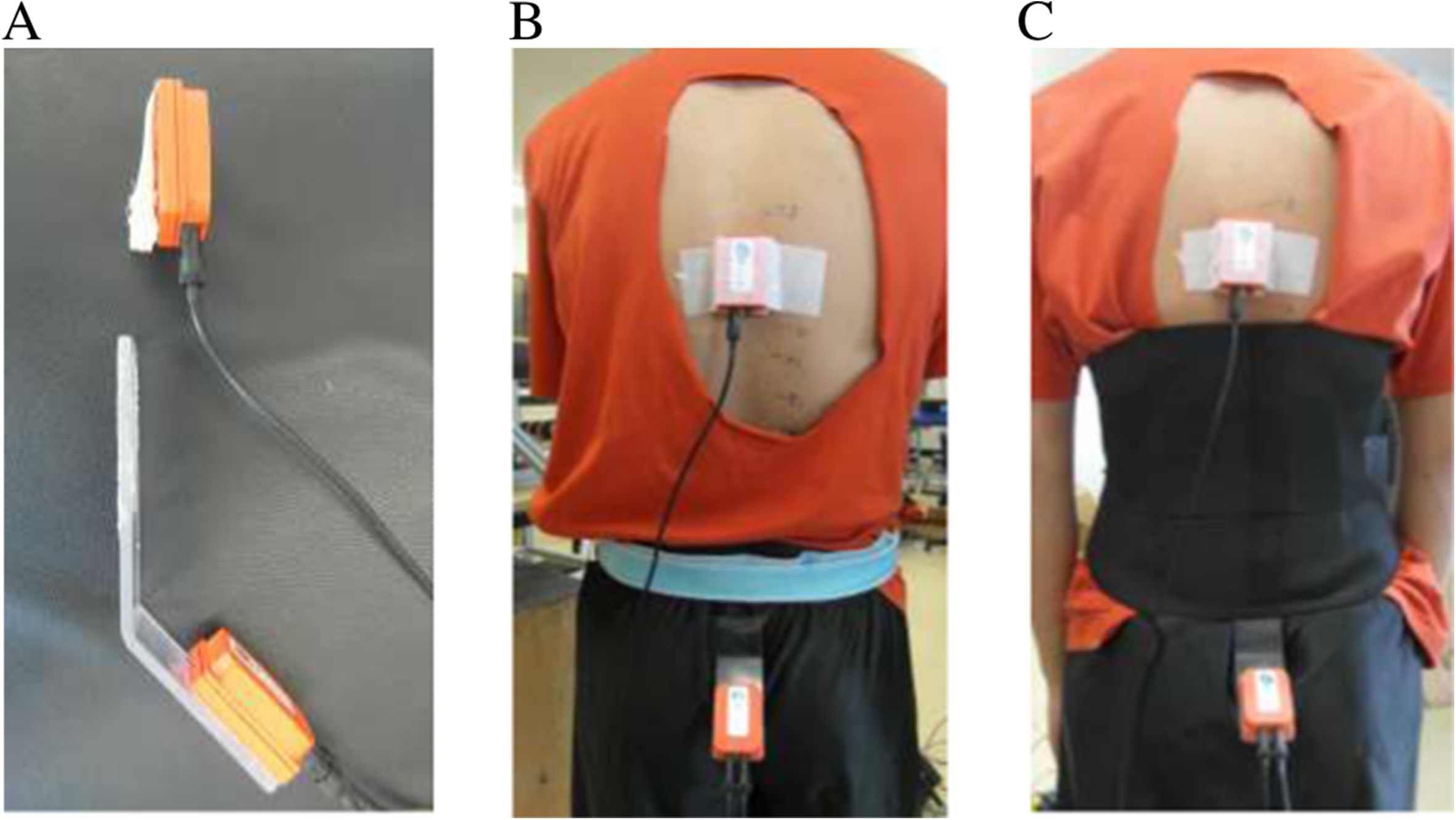

Supplement: Supplementary file 3 — Authors’ original file for figure 3 [file 12891_2014_2260_MOESM3_ESM.tif]

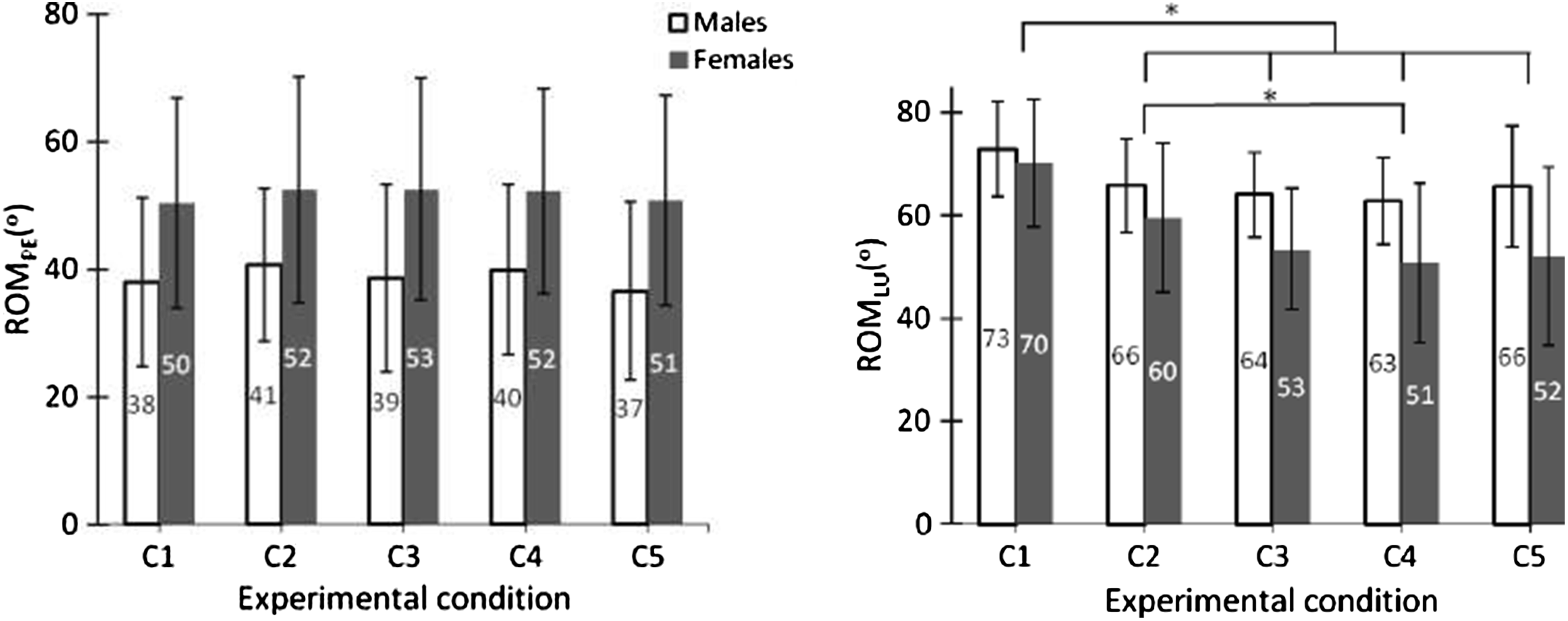

Supplement: Supplementary file 4 — Authors’ original file for figure 4 [file 12891_2014_2260_MOESM4_ESM.tif]

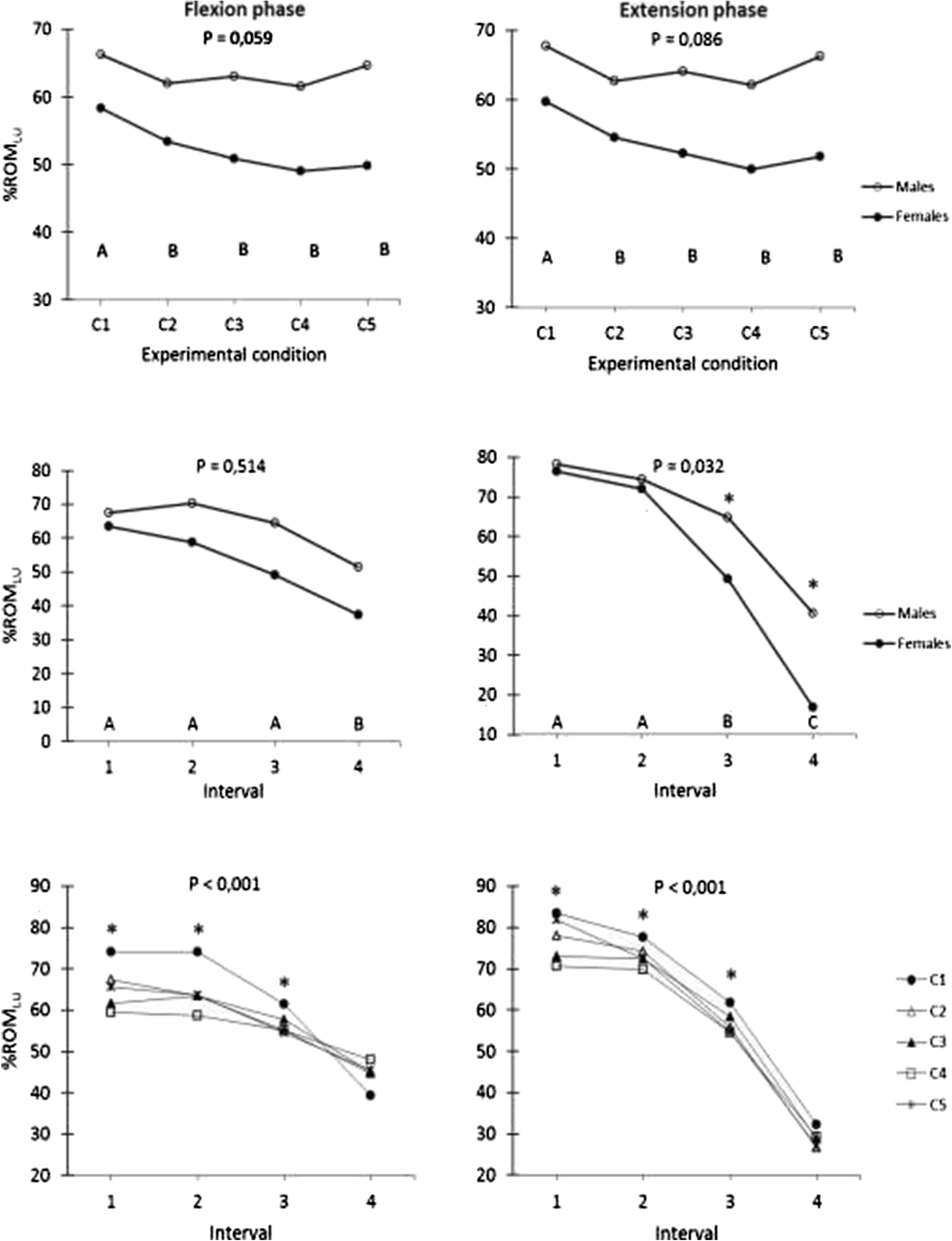

Supplement: Supplementary file 5 — Authors’ original file for figure 5 [file 12891_2014_2260_MOESM5_ESM.tif]

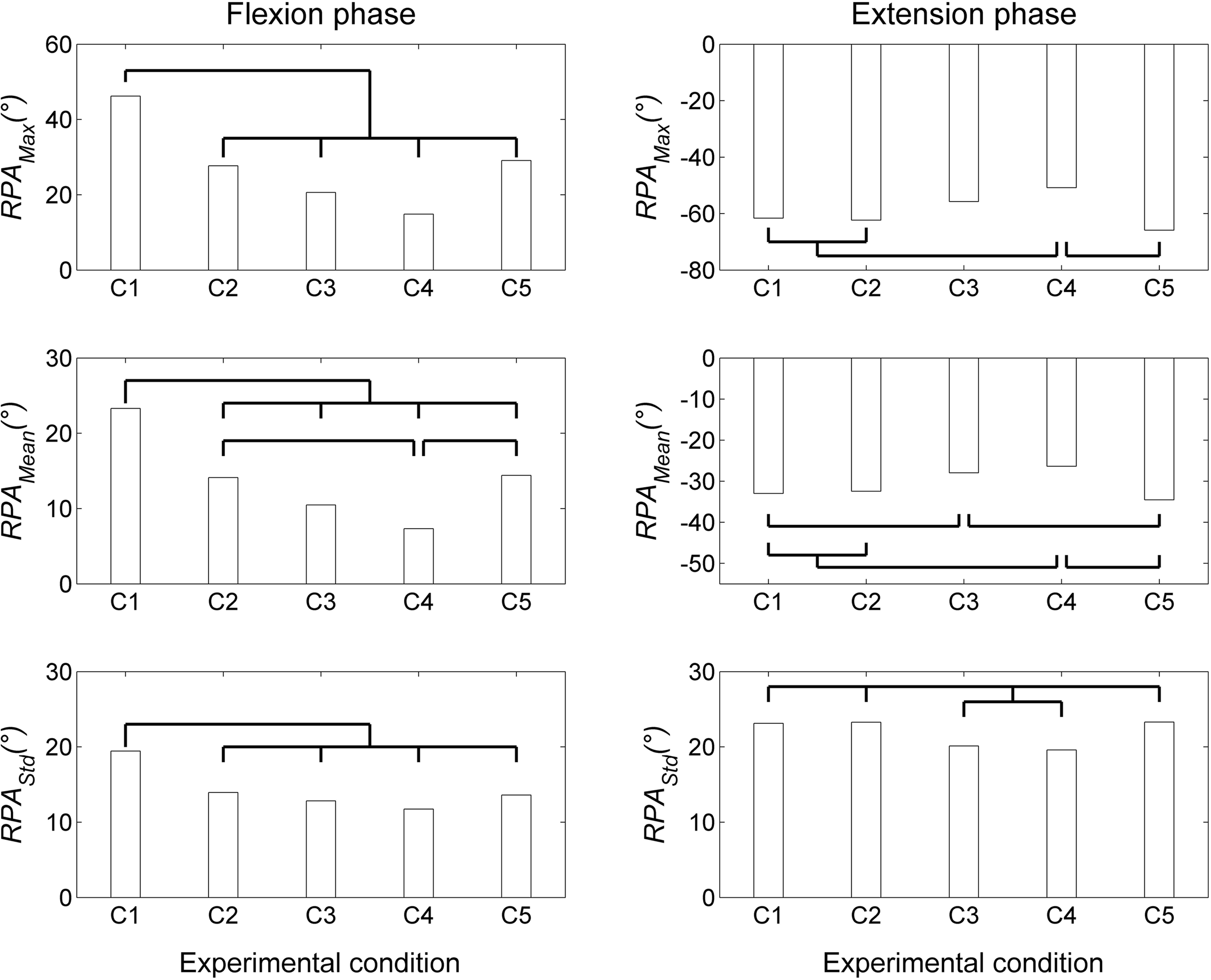

Supplement: Supplementary file 6 — Authors’ original file for figure 6 [file 12891_2014_2260_MOESM6_ESM.tif]
